# Cardiac Sarcoidosis-Induced Heart Failure

**DOI:** 10.7759/cureus.18685

**Published:** 2021-10-11

**Authors:** Michael W Figart, Krithika Suresh, David Bassilly, Jude Mugerwa

**Affiliations:** 1 Internal Medicine, Conemaugh Memorial Medical Center, Johnstown, USA; 2 Cardiology, Conemaugh Memorial Medical Center, Johnstown, USA

**Keywords:** cardiac sarcoidosis, heart failure, sarcoid induced heart failure, sarcoidosis, heart failure with reduced ejection fraction

## Abstract

Sarcoidosis is a common disease with the incidence of cardiac involvement varying. Cardiac sarcoidosis should be kept on the differential when young patients present with acute heart failure, conduction abnormalities or new arrhythmia. Cardiac involvement in sarcoidosis must be diagnosed early and treated aggressively. Here we present a patient who presented with shortness of breath and was found to have significant heart failure with reduced ejection fraction caused by sarcoidosis with cardiac involvement. She was treated with optimization of medical therapy for heart failure and eventually required implantable cardioverter defibrillator (ICD) placement.

## Introduction

Sarcoidosis is a rare inflammatory disorder of unknown etiology involving multi-organ systems. The cardiovascular system is affected rarely. A key feature of the disease is formation of noncaseating granulomas in the affected organs. In patients with systemic sarcoidosis, it is estimated that about 5% have symptomatic cardiac sarcoidosis (CS) [1]. Advances in diagnosis of cardiac involvement have come a long way from years past to now involving cardiac MRI and 18FDG-PET scanner. Corticosteroids have been found to be efficacious especially in patients who present with atrioventricular block and heart failure. Studies on immunosuppressive drugs have not been vigorously studied but they may have some benefit in treatment of sarcoidosis with cardiac involvement [2].

## Case presentation

**Figure 1:**
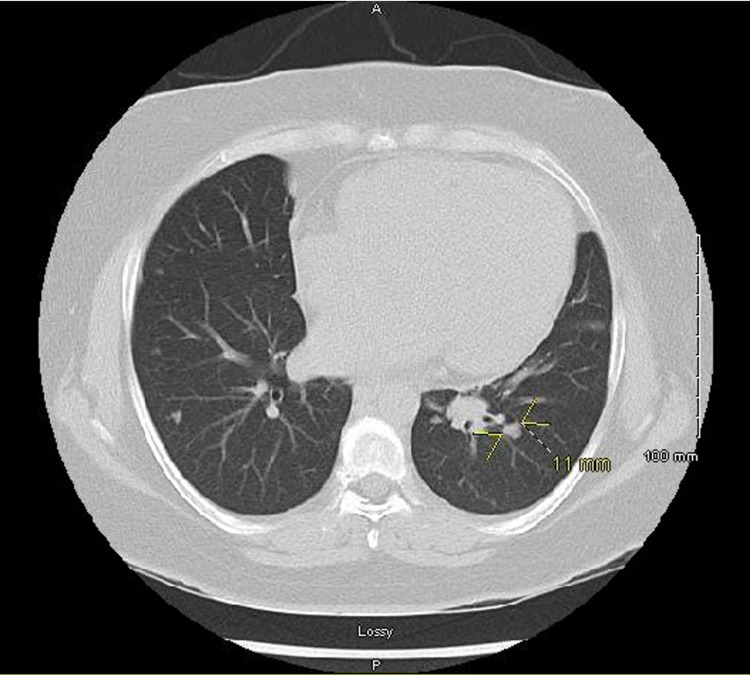
CT chest with scattered pulmonary nodules

A 40-year-old female with no prior medical history presented to her primary care physician (PCP) for shortness of breath (SOB) on exertion that had progressively worsened over the previous few months. She had a prior CT of her abdomen and it showed multiple pulmonary nodules at the lung bases. This led to further evaluation with CT chest which showed cardiomegaly and numerous scattered pulmonary nodules with the largest being 11 mm (Figure 1).

**Figure 2:**
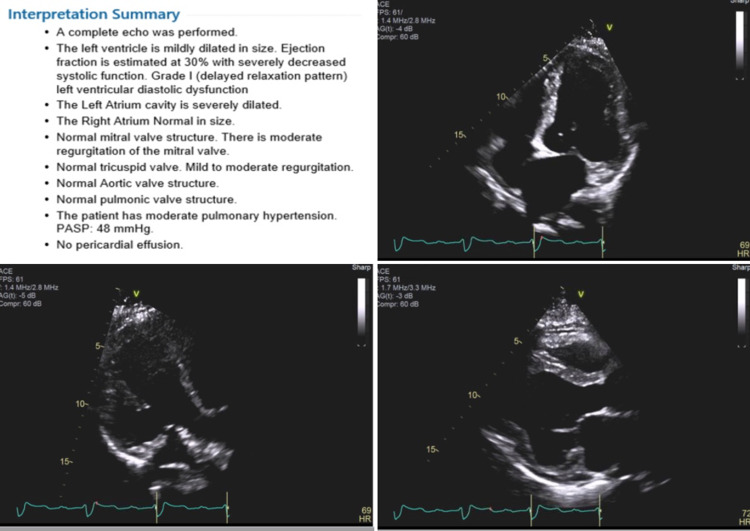
Echocardiogram

She then had an echocardiogram as an outpatient which showed an ejection fraction (EF) of 30%, mild mitral and tricuspid regurgitation and moderate pulmonary hypertension (Figure 2).

**Figure 3:**
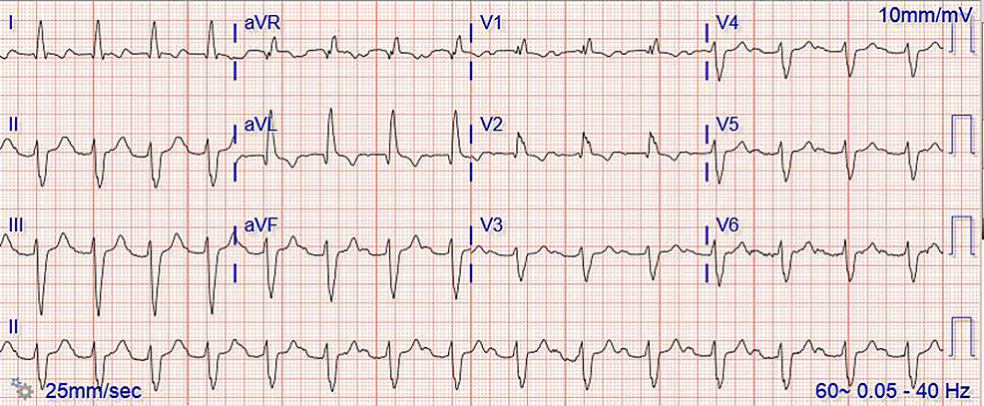
Electrocardiogram showing Right Bundle Branch Block and first-degree atrioventricular (AV) block

One month later she presented to the emergency department for worsening heart failure symptoms including worsening shortness of breath and lower extremity edema. She was symptomatic with New York Heart Association (NYHA) class III heart failure symptoms. Her electrocardiogram (EKG) showed right bundle branch block (RBBB) and first-degree atrioventricular (AV) block (Figure 3).

**Figure 4:**
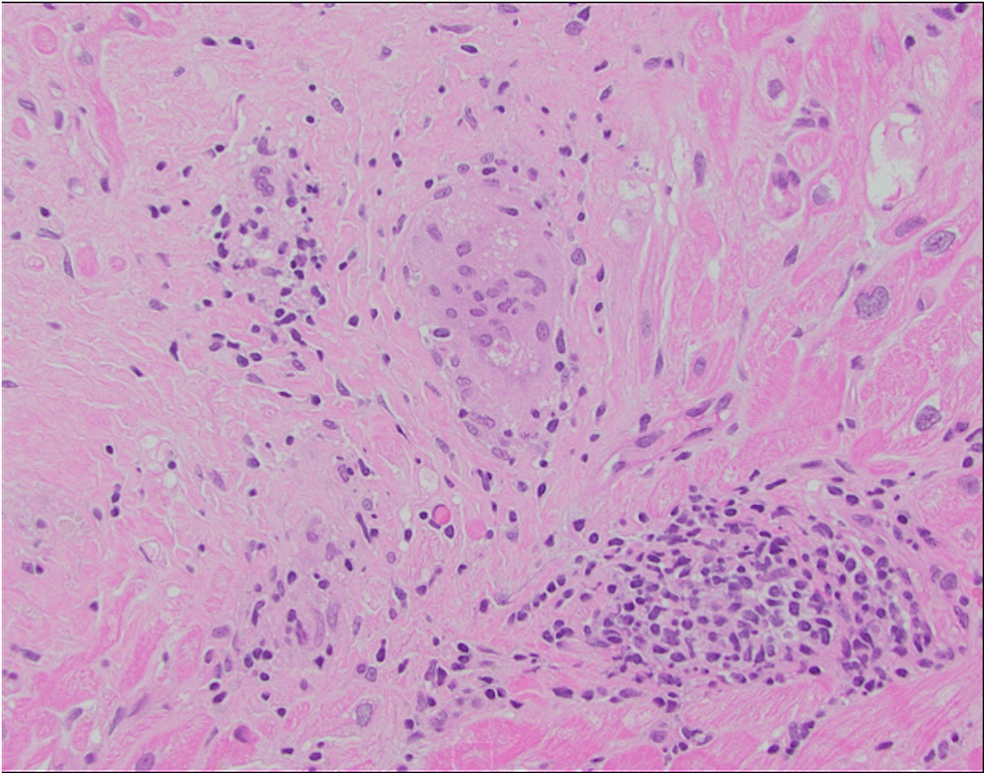
Cardiac biopsy showing dense interstitial fibrosis with non-necrotizing granulomas with mild inflammation composed of lymphocytes, macrophages and multinucleated giant cells, some containing asteroid bodies

Transthoracic echocardiogram was repeated, and her EF dropped to 14%. Cardiac MRI revealed severe cardiomyopathy with reduced EF estimated at 14% along with a patchy transmural fibrosis. A stress test did show abnormal left ventricular perfusion in a left circumflex distribution. Left heart catheterization was performed and showed no coronary artery disease. She then had a cardiac biopsy that was consistent with cardiac sarcoidosis showing dense interstitial fibrosis with non-necrotizing granulomas with mild inflammation composed of lymphocytes, macrophages and multinucleated giant cells, some containing asteroid bodies (Figure 4). She refused a Life Vest at this time due to it being uncomfortable. For her suspected sarcoidosis she was started on prolonged course of high dose prednisone which was slowly tapered. She was started on optimal medical therapy with loop diuretics, a beta blocker and an angiotensin-converting enzyme (ACE) inhibitor at maximum tolerated doses. Repeat echo at four months showed that her EF remained reduced at 20%. At this time, she did agree to a biventricular implantable cardioverter defibrillator (ICD). Methotrexate was later started as her sarcoidosis was refractory to glucocorticoids with leflunomide being added as well. 

## Discussion

Roughly 5% of patients with systemic sarcoidosis have clinically manifested cardiac involvement presenting with one or more combinations of ventricular arrhythmias, conduction abnormalities, and heart failure. About 20-25% of systemic sarcoidosis patients have asymptomatic cardiac involvement. The degree of left ventricular dysfunction is the most important predictor of prognosis among patients with clinically manifested CS [3]. Physical exam findings in isolated CS can be seen as a murmur (mitral regurgitation) due to dilated cardiomyopathy with or without papillary muscle dysfunction [4].

Patchy involvement with hypertrophied myocardium can lead to dilated cardiomyopathy due to enlargement of the ventricles. When diffuse involvement of the myocardium is seen, patients may present with heart failure due to dilatation of both ventricles to a significant extent. Involvement of the conduction system results in conduction abnormalities and varying degrees of AV block [5].

Complete AV block is the most common conduction system defect seen in patients with CS. Ventricular tachycardia/ventricular fibrillation is the second most common arrhythmia in patients who present with CS, both of which are associated with increased risk of sudden cardiac death [6]. 

Diagnosis of CS is challenging given the varied clinical manifestations and imperfect diagnostic techniques. Electrocardiography has low sensitivity and specificity for diagnosis of CS. Cardiac MRI accurately identifies areas of myocardial damage including edema and scar, primarily via late gadolinium enhancement (LGE) technique. Recently published guidelines acknowledge LGE on Cardiac MRI as a diagnostic criterion [7].

Cardiac involvement can be assessed with endomyocardial biopsy. However, due to patchy myocardial involvement in CS, diagnostic yield of endomyocardial biopsy is only about 20%. Thus, in most cases, diagnosis of CS is made when the patient demonstrates histologically confirmed extracardiac sarcoidosis and has non-histologic clues of CS [8]. However, this does not apply to isolated CS where endomyocardial biopsy still is a key diagnostic modality.

Oral corticosteroid is standard treatment. Immunosuppressive combination therapy with methotrexate or azathioprine is considered second-line treatment. A combination of leflunomide and mycophenolate mofetil can be contemplated if the patient does not tolerate first-line treatment due to side effects [9].

Early diagnosis and aggressive treatment of CS are essential in preventing the complications mentioned above, including sudden cardiac death [10]. 

## Conclusions

This case highlights the importance of including CS in the differential diagnosis of a young and otherwise healthy patient presenting with signs and symptoms of new onset heart failure and non-ischemic cardiomyopathy. Optimal medical management of heart failure should also be initiated. These patients are typically started on a prednisone taper and should strongly be considered for automated ICD when left ventricular dysfunction is present. In the case of persistent symptoms despite glucocorticoid therapy, immunosuppressive agents may be of benefit in symptom management.
